# Comparative Analysis of Annealing–Dissolution Techniques for Hollow Submicron Metal Oxide Fiber Synthesis

**DOI:** 10.3390/ma19020327

**Published:** 2026-01-14

**Authors:** Borislava Georgieva, Blagoy Spasov Blagoev, Albena Paskaleva, Kirilka Starbova, Nikolay Starbov, Ivalina Avramova, Peter Tzvetkov, Krastyo Buchkov, Vladimir Mehandzhiev

**Affiliations:** 1 Institute of Solid State Physics, Bulgarian Academy of Sciences, 72 Tsarigradsko Chaussee, 1784 Sofia, Bulgaria; b.georgiewa@abv.bg (B.G.); paskaleva@issp.bas.bg (A.P.); ilka_05@yahoo.com (K.S.); kikostar@mail.bg (N.S.); k.buchkov@hotmail.com (K.B.); vlado_bm@yahoo.com (V.M.); 2 Institute of Electronics, Bulgarian Academy of Sciences, 72 Tsarigradsko Chaussee, 1784 Sofia, Bulgaria; 3 National Centre of Excellence Mechatronics and Clean Technologies, 8 Kl. Ohridski, Bl. 8, BG-1000 Sofia, Bulgaria; 4 Institute of General and Inorganic Chemistry, Bulgarian Academy of Sciences, 1113 Sofia, Bulgaria; iva@svr.igic.bas.bg (I.A.); tzvetkov@svr.igic.bas.bg (P.T.)

**Keywords:** hollow submicron fibers, electrospinning, atomic layer deposition (ALD), metal oxide fibers, polyvinyl alcohol (PVA), Al_2_O_3_, ZnO, nanostructures, high-aspect-ratio (HAR), bubble-like structure

## Abstract

Double-shell ZnO/Al_2_O_3_ submicron hollow fibers were successfully fabricated through a combined electrospinning and atomic layer deposition (ALD) approach. Polyvinyl alcohol (PVA) fibers were first produced by electrospinning and subsequently coated with a conformal Al_2_O_3_ barrier layer via low-temperature ALD employing trimethylaluminum (TMA) and deionized (DI) H_2_O to preserve the integrity of the temperature-sensitive polymer core. The inner polymer was then removed using two different techniques—thermal annealing and water dissolution—to compare their effects on the fiber morphology. Finally, a functional ZnO layer was deposited by thermal ALD with diethylzinc (DEZ) and DI H_2_O. It was found that the polymer removal method critically determined the final structural and morphological characteristics of the fibers. Thermal annealing resulted in smooth, shrunken fibers, while water dissolution led to diameter expansion and the formation of a highly rough, bubble-like surface structure due to swelling-induced micro-cracking. The selection of the polymer removal method offers a precise and controllable route for tailoring the fiber morphology. The resulting high-aspect-ratio (HAR) structures, particularly the rough and expanded fibers, exhibit enhanced specific surface area, making them highly promising for applications in sensing, catalysis, and filtration.

## 1. Introduction

Nanostructures and nanomaterials are among the most rapidly developing areas of contemporary science. The field’s explosive growth is demonstrated by the sheer volume of literature: from 2003 to July 2024, the Chemical Abstracts Service (CAS) identified over three million journal articles, patents, preprints, and conference proceedings related to nanoscale topics [1,2]. This sustained interest stems from the unique properties of nanomaterials, which differ fundamentally from those of their bulk counterparts. Distinct characteristics such as electrical conductivity, catalytic activity, and optical behavior can be precisely tuned through nanotechnological processes [3]. This tunability extends to key physical and chemical parameters, including melting point, band gap, fluorescence, magnetic permeability, surface area, and chemical reactivity [2,4]. From both theoretical and practical standpoints, nanomaterials are compelling because they exhibit unique quantum effects arising from quantum confinement and offer a diverse range of applications, including catalysis, sensors, energy storage and conversion, photonics, environmental remediation, and biomedical devices [2].

Any material possessing at least one dimension within the 1–100 nm range is classified as a nanomaterial, which allows its categorization into 0D, 1D, 2D, or 3D structures. Among these, 3D nanomaterials (representing the most complex group) are high-aspect-ratio (HAR) structures built from nanostructured 0D, 1D, or 2D components. They account for over 85% of the “nanofamily” [5], owing to properties such as high specific surface area, porosity, controlled dimensionality, enhanced stability, and multifunctional integration. For many practical applications involving harsh operating conditions, particularly in strongly acidic or alkaline environments, chemically robust materials are essential [6]. Such robustness significantly broadens their applicability across diverse fields, including energy conversion and storage, sensing, catalysis, biomedicine, environmental remediation, and electronics [5,7].

To develop advanced 3D nanomaterials, researchers utilize a variety of chemical and physical methods, often in combination. The most prevalent synthesis techniques include hydrothermal synthesis, sol–gel processes, chemical vapor deposition (CVD), sputtering, spin coating, and spray pyrolysis [8,9,10]. Particularly promising is the integration of atomic layer deposition (ALD) with sacrificial templates that are subsequently removed [6]. This combined approach provides a versatile and robust pathway for fabricating a wide range of nanostructures. Examples of sacrificial templates include anodic aluminum oxide (AAO), carbon, silica spheres, biological structures, electrospun polymer fibers, polystyrene (PS) spheres, and photoresist polymers [6,11,12].

The combination of atomic layer deposition (ALD) and electrospinning is a powerful and flexible approach for fabricating high-aspect-ratio (HAR) nanostructures [13,14,15,16,17]. Initially, electrospinning is used to produce HAR polymer fiber scaffolds (e.g., PVA, PAN, PVP) with diameters ranging from tens of nanometers to several micrometers. ALD is then employed as a post-fabrication step to deposit thin, uniform, and pinhole-free inorganic films (e.g., Al_2_O_3_, ZnO, metal nanoparticles) onto these fibers, enabling precise control over the surface chemistry, composition, and structure of the final nanostructure. ALD is usually performed at low temperatures (e.g., 60 °C for PVA) to prevent polymer melting during deposition. However, such low temperatures often fall outside the ALD temperature window, which can compromise the quality of the films [11,12]. To obtain the hollow fiber structures, the inner polymer core is usually removed through high-temperature annealing (calcination). When conducted under an appropriate temperature regime, this process ensures complete removal of the polymer, resulting in smooth surfaces and enhanced crystallinity. An alternative, less studied method for polymer removal is dissolution [16,18,19,20,21]. While thermal annealing is well-established, the dissolution method remains comparatively underexplored, with limited available data. Fibers produced by dissolution often exhibit reduced lattice strain and increased surface roughness (e.g., coral-like structures [19]), which further enhances their specific surface area. Moreover, the dissolution method is essential when the hollow fiber structures are fabricated on flexible polymer substrates (e.g., PEN, PET, etc.) that would be damaged by thermal annealing. These unique advantages, together with the limited number of available comprehensive studies, motivated us to thoroughly investigate the polymer dissolution method. The comparison between the calcination and dissolution methods clearly highlights that the choice of preparation technique plays a decisive role in determining the resulting fiber morphology.

We present a method for synthesizing ZnO/Al_2_O_3_ double-shell hollow submicron fibers by combining electrospinning and atomic layer deposition (ALD). First, electrospun polyvinyl alcohol (PVA) fibers were coated with an Al_2_O_3_ layer using low-temperature (60 °C) ALD. The inner PVA core was then selectively removed using two different approaches: thermal annealing in air or dissolution in water. Finally, the ALO fibers were coated with a functional ZnO layer via thermal ALD performed within the appropriate ALD temperature window.

The resulting fibers were comprehensively characterized by scanning electron microscopy (SEM), X-ray diffraction (XRD), and X-ray photoelectron spectroscopy (XPS) to confirm their morphology and crystallographic properties.

## 2. Materials and Methods

### 2.1. Electrospinning

Submicron metal oxide fiber tubes were fabricated using electrospun polyvinyl alcohol (PVA) (home-made electrospinning equipment) fibers as sacrificial templates. The precursor solution was prepared by dissolving PVA grains (VALERUS, 93.5% purity, 7200 MW) in deionized (DI) water to obtain an 8 wt% solution. The mixture was stirred continuously at 60 °C for one hour until sufficient viscosity was achieved. Electrospinning was then performed at a flow rate of 1 mL/h under ambient conditions (29 kV, 24 °C) for six minutes, with fibers collected on 1 cm × 1 cm glass substrates (Deltalab, member of SCGP, Barcelona, Spain).

### 2.2. Low-Temperature ALD

The PVA fibers were subsequently coated with aluminum oxide (Al_2_O_3_, ALO) via atomic layer deposition (ALD) using a Beneq TFS-200 reactor (Beneq Oy, Espoo, Finland). Trimethylaluminum (Al_2_(CH_3_)_6_, TMA, Sigma-Aldrich, Merck Group, Burlington, MA, USA) and DI water served as the ALD precursors, with pulse and purge durations set at 300 ms and 5 s, respectively. To prevent degradation of the polymer templates, the reactor temperature was maintained at 60 °C during deposition, and a total of 150 ALD cycles were completed.

### 2.3. Removal of Polymer Core

Two approaches were employed to remove the inner polymer core: (i) high-temperature calcination at 500 °C in air for 24 h using a Carbolite horizontal tube furnace (Carbolite, Hope, Derbyshire, UK) and (ii) dissolution in deionized water (DI H_2_O) at varying temperatures and durations. The samples obtained via thermal annealing are denoted as series A, with detailed descriptions provided in our previous studies [11,12].

For the water dissolution experiments, the samples were placed in a beaker containing 500 mL of DI water, preheated to the desired temperature using a thermal bath equipped with a digital thermal controller (Omron E5CC, Kyoto, Japan). To investigate the optimal conditions for polymer dissolution, the temperature (*T*) and time duration (*t*) were systematically varied, as detailed in Table 1 (series D, E, F). The temperature deviation during each treatment was kept below 1 °C (Supplementary Materials). To preserve the integrity of the fiber mat, no stirring was applied during water treatment. Following dissolution, the samples were dried in air at room temperature on a cleanroom laboratory bench for 24 h. 

### 2.4. Thermal ALD

The resulting Al_2_O_3_ fiber tubes (series A, D, E, F) were subsequently coated with ZnO using atomic layer deposition (ALD), employing diethylzinc ((C_2_H_5_)_2_Zn, DEZ, Sigma-Aldrich, Merck Group, Burlington, MA, USA) and deionized water (DI H_2_O) as precursors. In the absence of the polymer core, the ZnO deposition was conducted at elevated temperatures (200 °C—series A0, D, E, F or 230 °C—series A3) to improve crystallinity and structural integrity. Both precursors were sequentially introduced into the reactor with a pulse duration of 300 ms, separated by a 5 s purge using high-purity nitrogen (Nitrogen 5.0). This ALD cycle was repeated 150 times.

### 2.5. Characterization Methods

The morphology, chemical composition, and crystallinity of the synthesized fiber tubes were characterized using scanning electron microscopy (SEM), X-ray photoelectron spectroscopy (XPS), and X-ray diffraction (XRD). These submicron tubes possess significant potential for gas sensor applications. The elevated specific surface area, along with the alteration in electrical resistance upon gas adsorption, is expected to markedly enhance the sensitivity of the sensor.

The morphology of the fiber surfaces was examined using a TESCAN LYRA SEM (TESCAN, Brno, Czech Republic). XRD measurements were performed on a Bruker D8 Advance Bragg–Brentano diffractometer (Bruker AXS, Karlsruhe, Germany) with a Cu Kα source (40 kV, 40 mA) and a LynxEye detector. Diffraction patterns were recorded over a 2Θ range of 10–70°, with a step size of 0.04° and a counting time of 4 s per step.

XPS measurements were performed using a VG ESCALAB MK II electron spectrometer (Thermo Fisher Scientific Inc., Waltham, MA, USA) equipped with an Al Kα X-ray source (1486.6 eV). The system operated at a base pressure of 1 × 10^−8^ Pa. The resolution was verified against the Ag 3d_5/2_ line, which demonstrated a FWHM of 1 eV at a 20 eV analyzer transmission energy. All binding energies were calibrated to the Au 4f_7/2_ peak (84.0 eV). Sample charging was corrected by referencing the adventitious carbon C 1s peak at 285 eV. The accuracy of the measured binding energy was ±0.2 eV.

Data analysis utilized the Shirley background subtraction technique. The O1s spectrum was deconvoluted using a single component peak. Position and FWHM constraints were both set to ±0.5 eV. XPSPEAK41 was used for peak fitting, ensuring that the goodness-of-fit criterion, the chi factor (χ), was equal to 1.

## 3. Results and Discussion

ZnO/Al_2_O_3_ (ZnO/ALO) double-shell hollow submicron fibers were synthesized on glass substrates through a four-step process: (1) electrospinning of polyvinyl alcohol (PVA) fibers onto the glass substrate; (2) deposition of an (ALO) film onto the PVA templates using low-temperature atomic layer deposition (LT-ALD) to preserve the polymer’s integrity; (3) removal of the inner polymer core by either water dissolution or high-temperature annealing; (4) completion of the double-shell structure by depositing ZnO onto the hollow ALO fibers via thermal ALD (T-ALD). In contrast to standard electrospinning–ALD protocols, our approach utilizes two distinct ALD steps. The first (LT-ALD) ensures the structural integrity of the polymer template, while the second (T-ALD), performed after polymer removal, is essential for improving the ZnO microstructure, composition, and crystallinity. A detailed description of this modified approach is provided elsewhere [11,12].

**Figure 1:**
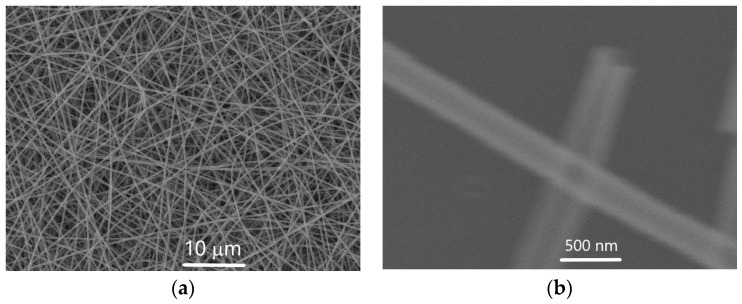
SEM analysis of ZnO/ALO hollow fibers obtained by thermal annealing (series A) with (**a**) 5.00 kx and (**b**) 100.00 kx magnification respectively.

**Figure 2:**
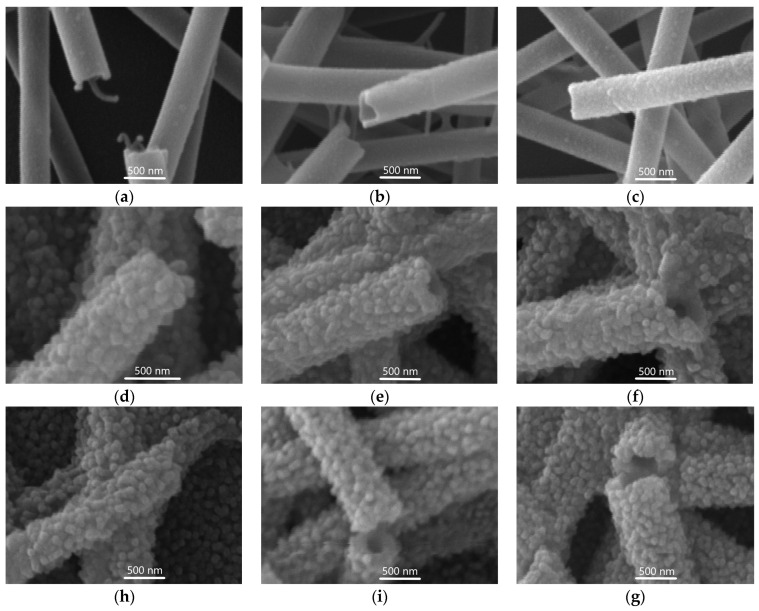
SEM analysis of ZnO/ALO hollow fibers obtained by dissolution (Table 1): (**a**) D1; (**b**) D2; (**c**) D3; (**d**) E1; (**e**) E2; (**f**) E3; (**h**) F1; (**i**) F2; (**g**) F3.

The synthesized high-aspect-ratio (HAR) structures consist of well-defined hollow fibers, as confirmed by scanning electron microscopy (SEM). The ZnO/ALO fibers obtained via thermal annealing exhibit a smooth, continuous, and uniform surface (Figure 1), whereas those produced through water-assisted polymer removal display a comparatively rougher and less uniform surface (Figure 2).

Furthermore, the morphology of the dissolved fibers was strongly dependent on the treatment parameters: increasing the water temperature and duration resulted in higher surface roughness. At 50 °C and 80 °C, the fiber surfaces developed a distinctive bubble-like structure with clearly visible grains. The fiber surface is densely covered by grains with a mean diameter of approximately 100 nm. SEM analysis of the fibers obtained by dissolution at 30 °C reveals the initial stages of grain formation (Figure 2a–c). The dissolution conditions determined the evolution of the surface morphology: longer durations led to a gradual increase in roughness and total surface coverage. Notably, at higher temperatures, the characteristic bubble-like structure fully developed within 30 min (Figure 2d,h). Further extension of the dissolution duration at elevated temperatures (50 °C and above) did not result in significant changes to the final surface morphology.

**Figure 3:**
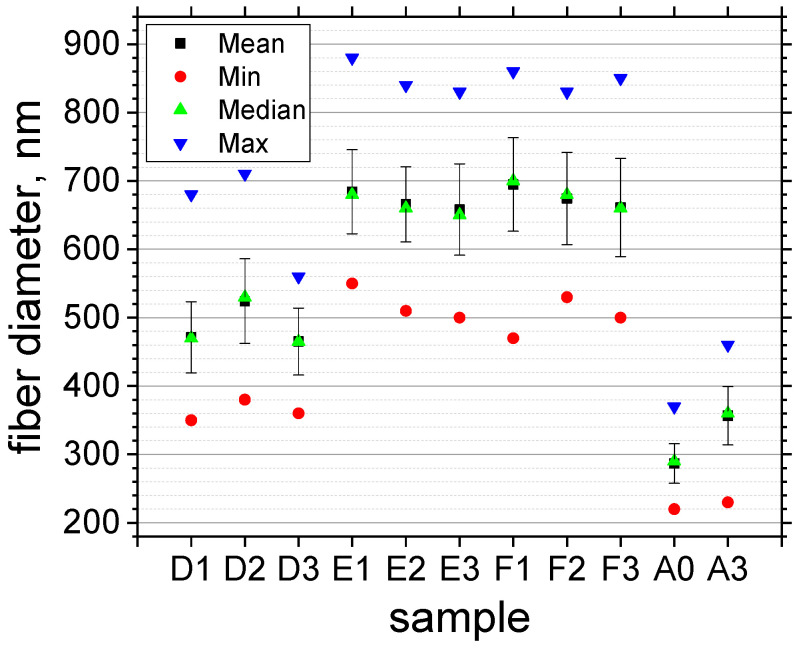
Statistics of ZnO/ALO hollow fiber diameters.

A quantitative analysis of fiber thickness was conducted using SEM images acquired at 20 kV and 5000× magnification. Descriptive statistical analysis was conducted to determine the mean, standard deviation, minimum, median, and maximum fiber thickness for all samples. The thickness distribution, obtained from 150 measurements per sample, was found to follow a normal distribution (see Supplementary Materials). The mean fiber thickness and corresponding standard deviations for each sample are presented in Figure 3. Fibers from which the polymer core was removed via thermal annealing (samples A0 and A3) exhibited significantly reduced diameters compared to fibers prepared by water dissolution (samples D, E, and F). This reduction is attributed to the known shrinkage of the ALO/PVA structure during annealing at 500 °C in air [11,12]. Moreover, the elevated ZnO deposition temperature for sample A3 (230 °C) compared to A0 (200 °C) resulted in an increased ALD growth rate, yielding a thicker ZnO layer for the same number of cycles. Conversely, immersion of ALO/PVA fibers in water induces swelling of the polymer core, leading to overall fiber expansion. This phenomenon is consistent with the mechanism proposed by Heikkilä et al. [19], whereby polymer swelling generates microcracks in the Al_2_O_3_ tube walls. As the polymer core swells, it exerts outward radial stress on the rigid Al_2_O_3_ shell. Higher temperatures accelerate this swelling, as evidenced by the greater initial diameter increase observed in Series E and F (Figure 3). Once the internal stress exceeds the mechanical strength of the shell, localized micro-cracks and rupture points form. These microcracks and pores facilitate solvent penetration into the tube and the subsequent diffusion of the dissolved polymer outwards. The subsequent ALD of ZnO onto this rough, micro-cracked surface results in the formation of the observed bubble-like morphology. Crucially, the initial surface roughness and micro-strain in the ALO walls directly affect the grain density in the subsequently deposited ZnO film, thereby providing a quantitative metric for monitoring the evolution of the deposition process. Our statistical analysis of fiber thickness and surface morphology provides quantitative evidence for this swelling-induced expansion and cracking mechanism. This process is highly sensitive to temperature. For Sample D (water dissolution at 30 °C), the fiber thickness is significantly greater than that of the annealed structures (Sample A series) but remains considerably lower than in the E and F series, which were treated at higher temperatures. At 30 °C (Series D), the micro-crack formation is slow and isolated: during the first 60 min, the PVA core swells and the tube expands (Figure 3, D1 and D2). The formation of only a few isolated cracks results in a low density of nanoscale ZnO grains (Figure 2a,b). After 120 min of treatment, a greater number of pores are generated, allowing more polymer to dissolve and diffuse out, while the tube relaxes through shrinking (Figure 2c and Figure 3, sample D3). At 50 °C and 70 °C (Series E and F; Figure 3), the accelerated swelling rapidly leads to fully developed and widely distributed micro-cracks across the surface within 30 min, giving rise to the characteristic bubble-like morphology (Figure 2d–g). Once the micro-cracks open, the dissolved polymer is released, and the internal pressure drops. The thin shell then undergoes structural relaxation, tending toward a lower-energy state. Longer treatment times facilitate more complete polymer removal and maximum relaxation, resulting in a measurable reduction in fiber thickness and stabilization of the morphology observed in the later stages of Series D, E, and F (Figure 3).

**Figure 4:**
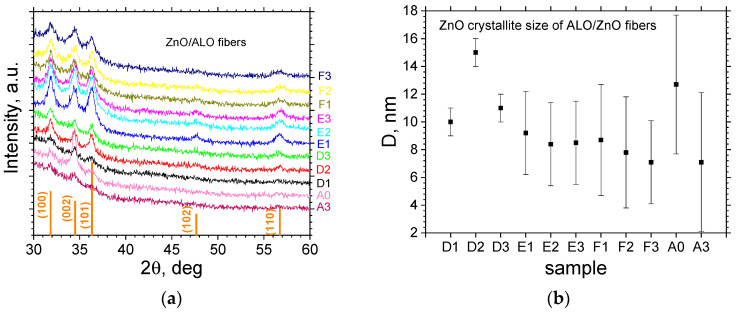
X-ray diffraction analysis of ZnO/ALO hollow fibers. (**a**) XRD patterns of the ZnO/ALO hollow fibers obtained by water dissolution (Series D, E, F) and thermal annealing (Series A). (**b**) Calculated ZnO crystallite sizes for the corresponding samples.

The X-ray Diffraction (XRD) analysis confirmed the formation of a polycrystalline hexagonal phase with a wurtzite-type structure (Figure 4). The XRD patterns are highly consistent with the surface morphologies discussed earlier. For the ZnO/ALO hollow fibers obtained by thermal annealing (Series A), the reflections with *hkl* indexes (002) show higher intensity in comparison to the bulk material. This indicates that the ZnO film grew smoothly with a predominantly *c*-axis orientation perpendicular to the fiber surface. Notably, Sample A0, grown at 200 °C, exhibits superior *c*-oriented crystallinity and larger crystallites compared to Sample A3, grown at 230 °C. This decrease in crystallinity at 230 °C is attributed to the ZnO growth shifting into a mixed ALD–CVD regime beyond the ALD window [12]. In contrast, samples obtained by dissolution (Series D, E, F) exhibit a rough surface morphology corresponding to the bubble-like structure. The corresponding diffraction patterns show strong signals for the (100), (002), and (101) reflections, but also feature weaker signals from the (102) and (110) planes. This overall pattern suggests the presence of crystallites with varied orientation, consistent with the highly disordered and rough surface morphology. XRD analysis (Figure 4a) clearly shows a higher diffraction peak intensity for hollow fibers obtained by water dissolution (Series D, E, F) compared to those obtained by thermal annealing (Series A). This is primarily attributed to a greater total amount of ZnO present in the dissolution-treated fibers. As the ALD parameters and substrate area (1 cm^2^ glass) were identical for all samples, this difference stems from the increased surface roughness created by the dissolution process. Since ALD is a self-limiting process that achieves conformal coverage (confirmed by the long pulse duration: 300 ms pulse, 5 s purge), the enhanced surface area of the bubble-like structures inherently results in the deposition of a larger overall ZnO mass. The highest peak intensity is observed in Series E, indicating optimal dissolution parameters. A subsequent decrease in the peak intensity in Series F (higher dissolution temperature) suggests a possible degradation in crystallinity or structural integrity. This finding regarding the saturation and optimization of the dissolving parameters in Series E is consistent with the SEM analysis discussed previously. A parallel trend is observed between the crystallite size and the fiber diameter across Series D, E, and F (Figure 3 and Figure 4b). This is explained by the swelling-induced expansion mechanism: during fiber expansion, the size and density of micro-cracks, pores, defects, and surface strain increase. These morphological features and associated stresses partially relax during the subsequent shrinking phase following polymer removal. Crucially, these micro-cracks and defects serve as nucleation sites for the ALD film; consequently, larger initial defects directly promote the formation of larger ZnO crystallites.

**Figure 5:**
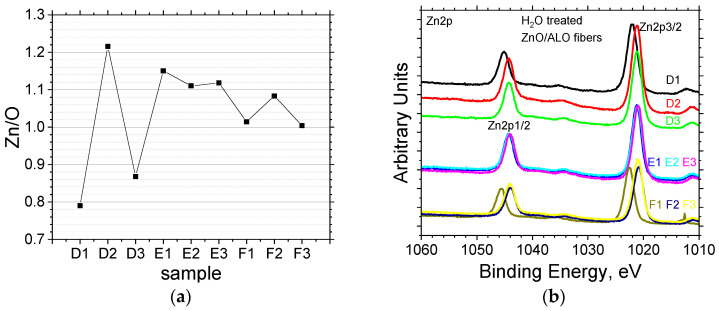
XPS analysis of ZnO/ALO hollow fibers obtained by water dissolution (Series D, E, F). (**a**) Zn/O atomic ratio calculated from the integrated peak areas of the Zn2p and O1s core level signals. (**b**) High-resolution Zn2p spectrum showing the characteristic Zn2p_3/2_ and Zn2p_1/2_ spin–orbit components, confirming the Zn^2+^ chemical state.

The elemental composition and chemical state of the fiber surface were analyzed by X-ray Photoelectron Spectroscopy (XPS) for samples prepared via water dissolution (Table 2). At the investigated surface depth (~3–5 nm), the core levels Zn2p, O1s, and C1s were successfully detected. The atomic concentrations of the detected elements (carbon, zinc, and oxygen) were calculated from the integrated peak areas of the C1s, Zn2p, and O1s core level signals. The concentration of C in these samples is attributed primarily to surface adventitious carbon contamination [11] and, secondarily, to any remaining carbon from the dissolution process. Except for the sample D1, where the carbon concentration exceeds 20%, for all other samples it is in the range 12–19%, showing no clear dependence on the dissolution temperature or duration. In contrast, the O and Zn atomic concentrations show a clear variation across the samples, oscillating between 34% and 47%. Importantly, the Zn/O ratio exhibits a strong trend: increasing the temperature and duration of the water treatment causes the ratio to approach unity (1.0) (Figure 5a), indicating that the surface composition moves closer to stoichiometric ZnO (1:1 ratio). This suggests that more complete removal of non-stoichiometric phases or surface-adsorbed species occurs under harsher dissolution conditions.

The Zn2p photoelectron spectrum (Figure 5b) shows the expected Zn2p1/2 and Zn2p3/2 doublet split by ~23 eV. We observed a general shift in these peaks toward lower binding energy with increasing treatment duration. In Series E and F, this shift aligns with previous reports that link lower binding energy to smaller ZnO crystallite sizes [22]. The crystallite size reduction is attributed to the creation of broken bonds. In the bulk material, zinc cations (Zn^2+^) are tetrahedrally coordinated with four oxygen ions (O^2−^), corresponding to zero broken bonds. An increase in the number of broken bonds is directly proportional to the number of oxygen ions removed from the lattice surrounding a specific zinc cation. As the number of broken bonds increases, the charge transfer from the zinc ion to neighboring oxygen ions decreases, resulting in a lower BE of the Zn core electrons. Therefore, the lowering of the BE for a specific Zn peak can be definitively assigned to zinc ions that possess broken bonds [22]. However, Sample D1 (minimal processing) is a notable exception. Although its small crystallite size (D1, Figure 4b) predicts a low-energy shift, D1’s highest retained C surface contamination (Table 2) induces a counteracting shift toward higher binding energy [23,24]. This observation suggests a competition where the high carbon content significantly alters the local electronic environment, thus dominating and reversing the expected binding energy shift dictated by crystallite size. The minimal shift observed in the Zn2p peaks for Series E indicates a limited variation in the concentration of broken bonds. This structural consistency suggests a greater stability during the preparation process and confirms the previously mentioned optimal technological regime for this specific series.

**Figure 6:**
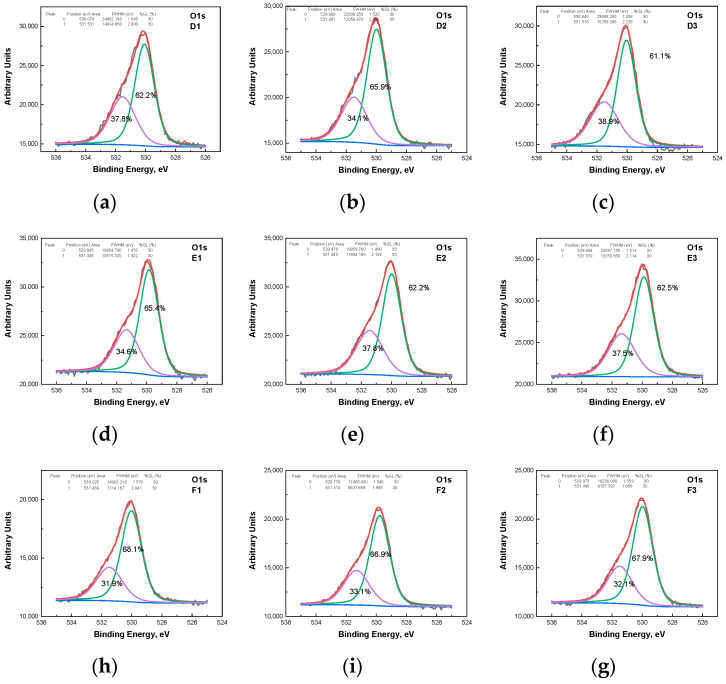
XPS deconvolution of the O1s core level. Deconvoluted O1s spectra for the ZnO/ALO hollow fibers obtained by water dissolution: (**a**) D1; (**b**) D2; (**c**) D3; (**d**) E1; (**e**) E2; (**f**) E3; (**h**) F1; (**i**) F2; (**g**) F3.

The deconvolution of the O1s photoelectron spectra (Figure 6) reveals two primary Gaussian components: (1) a peak centered around 530 eV, characteristic of lattice oxygen within the stoichiometric ZnO wurtzite structure; and (2) a peak at approximately 531.5 eV, attributed to oxygen vacancies or defects (non-stoichiometric oxygen). Although slight deviations in the peak position were noted for both components, no clear correlation with the water treatment parameters was established (Supplementary Materials). Crucially, the integrated peak area corresponding to the ZnO lattice (~530 eV) slightly decreases relative to the non-stoichiometric oxygen peak (~531.5 eV) as the treatment time increases (Supplementary Materials). This finding is consistent with the preceding discussion: increased treatment duration leads to a decrease in the crystallite size of ZnO, which in turn results in an increase in the number of broken bonds and, consequently, a higher number of oxygen ions removed from the lattice points. This directly accounts for the decreasing area of the ZnO lattice peak. Further details on the O1s deconvolution spectra are provided in our previous work [11,12].

## 4. Conclusions

We successfully fabricated hollow ZnO/ALO submicron fibers using a combined electrospinning and atomic layer deposition approach. The inner polymer core was removed using two distinct methods: thermal annealing and dissolution in deionized water. In thermal annealing (Series A), the fibers exhibited a smooth surface morphology and a shrunken fiber diameter relative to the initial polymer template. In water dissolution (Series D, E, F), the fibers displayed a characteristic rough, bubble-like surface structure and a swelling-induced expansion relative to the initial template. Increasing the temperature and duration of the water dissolution process accelerates polymer removal and leads to a simultaneous increase in fiber thickness, ZnO grain size, and grain density. The performed analysis revealed a critical processing window: low dissolution temperatures are insufficient for complete polymer core removal, whereas higher temperatures induce structural imperfections. Crucially, saturation of these key characteristics is reached at 50 °C and 60 min of treatment, establishing the optimal processing parameters. The distinct, roughness-enhanced, bubble-like structure of the dissolution-derived fibers results in a significantly increased specific surface area. These hollow ZnO/ALO high-aspect-ratio (HAR) structures, particularly those exhibiting the unique bubble-like morphology, hold great promise for future applications in fields such as catalysis, sensing, water purification, and other areas requiring enhanced surface activity.

The thermal annealing method yields structures (Series A) suitable for applications requiring mechanical robustness and precise dimensions, whereas the water dissolution method, particularly under optimal conditions (Series E), produces surface-enhanced fibers ideal for catalysis, sensing, and adsorption due to their high specific surface area and density of active sites. Moreover, the hollow fibers obtained via the dissolution method exhibit reduced mechanical stability. This limitation suggests that scaling up to larger or more complex substrates may be challenging and warrants further investigation. Consequently, a key direction for future work is the investigation of polymer substrates as alternatives to the current glass substrate.

## Figures and Tables

**Table 1:** Parameters for water-dissolving polymer core.

**Sample**	D1	D2	D3	E1	E2	E3	F1	F2	F3
***T*, °C**	30	30	30	50	50	50	80	80	80
***t*, min**	30	60	120	30	60	120	30	60	120

**Table 2:** Elemental concentrations extracted from XPS analysis. Concentrations of C, O, and Zn core levels for the ZnO/ALO hollow fiber samples obtained via water dissolution (Series D, E, F).

Sample	Concentration, %		
	C	O	Zn
D1	22.8	43.1	34.1
D2	13.8	38.9	47.3
D3	16.1	44.9	39
E1	14.6	39.7	45.7
E2	16.2	39.7	44.1
E3	12.2	41.5	46.3
F1	19.2	40.1	40.7
F2	14.3	41.1	44.6
F3	16.4	41.7	41.9

## Data Availability

The original contributions presented in this study are included in the article/Supplementary Material. Further inquiries can be directed to the corresponding author.

## Supplementary Materials

The following supporting information can be downloaded at: https://www.mdpi.com/article/10.3390/ma19020327/s1, Figure S1: Temperature deviation of water dissolution of PVA in ZnO/Al_2_O_3_ fibers at different temperature treatments: (a) 30 °C; (b) 50 °C; (c) 80 °C. Figure S2: Thickness distribution of ZnO/Al_2_O_3_ hollow fibers obtained by electrospinning, ALD and polymer core removal by water dissolving at different parameters. Water temperature and dissolving duration were: (a) 30 ℃, 30 min (D1); (b) 30 ℃, 60 min (D2); (c) 30 ℃, 120 min (D3); (d) 50 ℃, 30 min (E1); (e) 50 ℃, 60 min (E2); (f) 50 ℃, 120 min (E3); 80 ℃, 30 min (F1); 80 ℃, 60 min (F2); 80 ℃, 120 min (F3). The ZnO film is deposited by ALD at 200 ℃ after polymer removal. Figure S3: Thickness distribution of ZnO/Al_2_O_3_ hollow fibers obtained by electrospinning, ALD and polymer core removal by thermal annealing at 500 ℃ for 24 h in air. The ZnO film is deposited by ALD at (a) 200 ℃ or (b) 230 ℃ after polymer removal. Figure S4: XPS spectrum of the C1s core level. The main component is the C-C peak, typically observed at a binding energy of ~285 eV. Figure S5: Analysis of deconvoluted O1s spectra for ZnO/ALO hollow fibers. Plots showing the quantitative parameters extracted for the two main oxygen components: (a) peak position, (b) full width at half maximum (FWHM), and (c) integrated peak area.
