# Supplementary material for: Comparative Analysis of Annealing–Dissolution Techniques for Hollow Submicron Metal Oxide Fiber Synthesis
Source: Materials (Basel). 2026 Jan 14;19(2):327. doi: 10.3390/ma19020327 (PMC12843503; doi:10.3390/ma19020327)
Supplement: Supplementary file 1 [file materials-19-00327-s001.zip › materials-4004097-supplementary.pdf]

Supplementary Materials

Comparative Analysis of Annealing–Dissolution Techniques for Hollow Submicron Metal Oxide Fiber Synthesis

Borislava Georgieva [1,2,†], Blagoy Spasov Blagoev [1,*,†], Albena Paskaleva [1,3], Kirilka Starbova [1], Nikolay Starbov [1], Ivalina Avramova [4], Peter Tzvetkov [4], Krastyo Buchkov [1] and Vladimir Mehandzhiev [1]

[1] Institute of Solid State Physics, Bulgarian Academy of Sciences, 72 Tsarigradsko Chaussee, 1784 Sofia, Bulgaria; b.georgiewa@abv.bg (B.G.); blago_sb@yahoo.com (B.S.B.); paskaleva@issp.bas.bg (A.P.); ilka_05@yahoo.com (K.S.); kikostar@mail.bg (N.S.); k.buchkov@hotmail.com (K.B.); vlado_bm@yahoo.com (V.M.)
[2] Institute of Electronics, Bulgarian Academy of Sciences, 72 Tsarigradsko Chaussee, 1784 Sofia, Bulgaria; b.georgiewa@abv.bg (B.G.)
[3] "National Centre of Excellence Mechatronics and Clean Technologies", Kl. Ohridski Blvd, 8, Bl. 8, BG-1000 Sofia, Bulgaria
[4] Institute of General and Inorganic Chemistry, Bulgarian Academy of Sciences, 1113 Sofia, Bulgaria; iva@svr.igic.bas.bg (I.A.); tzvetkov@svr.igic.bas.bg (P.T.)
[*] Correspondence: blago_sb@yahoo.com; blago@issp.bas.bg
[†] These authors contributed equally to this work.

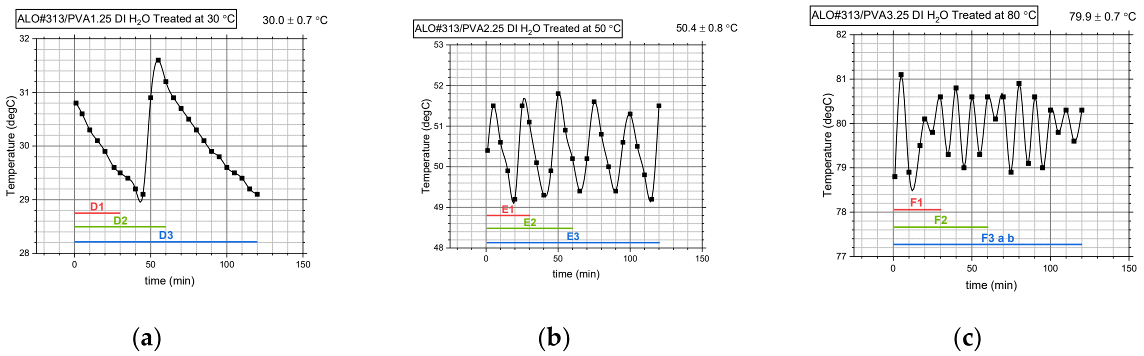

Figure S1. Temperature deviation of water dissolution of PVA in ZnO/Al_2O_3 fibers at different temperature treatments: (**a**) 30 °C; (**b**) 50 °C; (**c**) 80 °C.

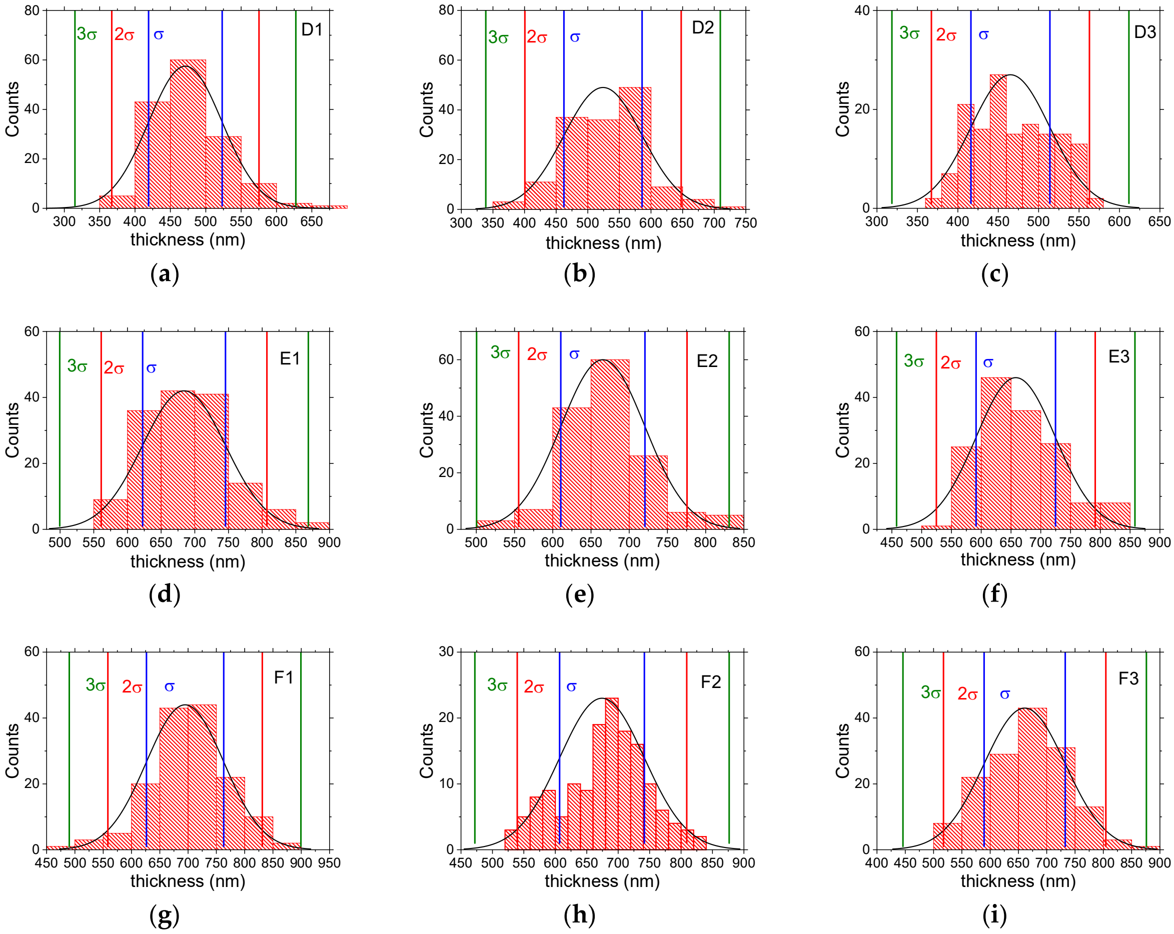

Figure S2. Thickness distribution of ZnO/Al_2O_3 hollow fibers obtained by electrospinning, ALD and polymer core removal by water dissolving at different parameters. Water temperature and dissolving duration were: (a) 30 °C, 30 min (D1); (b) 30 °C, 60 min (D2); (c) 30 °C, 120 min (D3); (d) 50 °C, 30 min (E1); (e)

50 °C, 60 min (E2); (f) 50 °C, 120 min (E3); (g) 80 °C, 30 min (F1); (h) 80 °C, 60 min (F2); (i) 80 °C, 120 min (F3). The ZnO film is deposited by ALD at 200 °C after polymer removal.

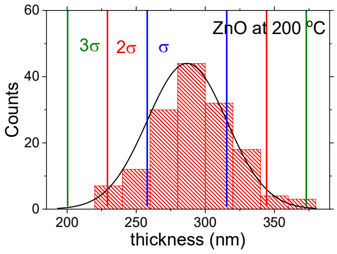

(a)

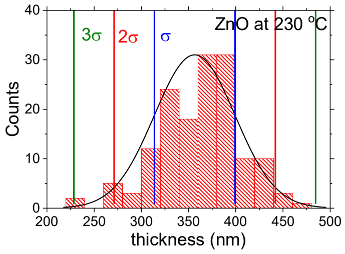

(b)

Figure S3. Thickness distribution of ZnO/Al_2O_3 hollow fibers obtained by electrospinning, ALD and polymer core removal by thermal annealing at 500 °C for 24 h in air. The ZnO film is deposited by ALD at (a) 200 °C or (b) 230 °C after polymer removal.

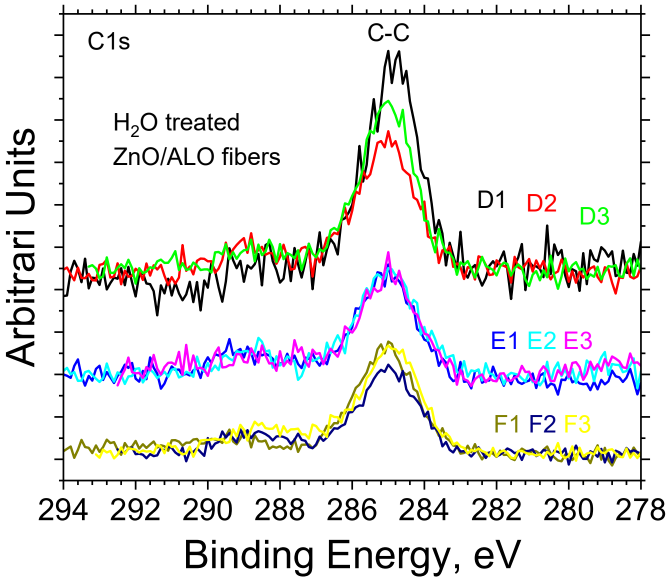

Figure S4. XPS spectrum of the C1s core level. The main component is the C-C peak, typically observed at a binding energy of ~285 eV.

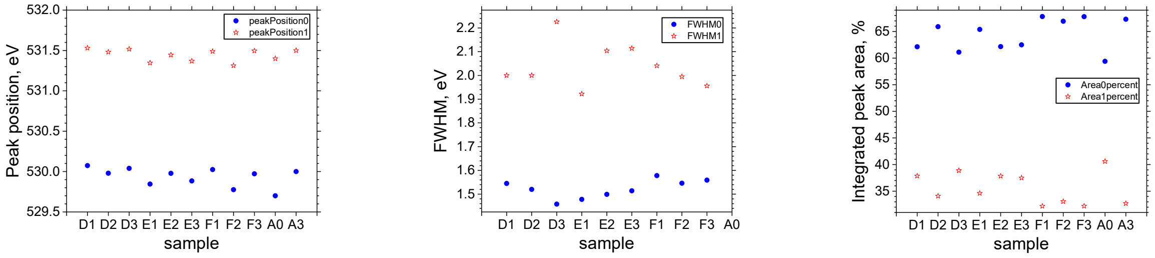

(**a**) (**b**) (**c**)

Figure S5. Analysis of deconvoluted O1s spectra for ZnO/ALO hollow fibers. Plots showing the quantitative parameters extracted for the two main oxygen components: (**a**) peak position, (**b**) full width at half maximum (FWHM), and (**c**) integrated peak area.
